# Association of the MicroRNA-146a SNP rs2910164 with Ischemic Stroke Incidence and Prognosis in a Chinese Population

**DOI:** 10.3390/ijms17050660

**Published:** 2016-05-05

**Authors:** Jiao-Yan Qu, Jie Xi, Yin-Hui Zhang, Chan-Na Zhang, Li Song, Yan Song, Ru-Tai Hui, Jing-Zhou Chen

**Affiliations:** 1 Sino-German Laboratory for Molecular Medicine, State Key Laboratory of Cardiovascular Disease, National Center for Cardiovascular Diseases, Fuwai Hospital, Chinese Academy of Medical Sciences, 167 Beilishilu, Beijing 100037, China; qujiaoyan1989@163.com (J.-Y.Q.); xijie226688@163.com (J.X.); zyh0863@163.com (Y.-H.Z.); china800908@hotmail.com (C.-N.Z.); lisong97@aliyun.com (L.S.); yansong1234@hotmail.com (Y.S.); huirutai@sglab.org (R.-T.H.); 2 Department of Hematology, Beijing Luhe Hospital Affiliated to Capital Medical University, Beijing 101149, China

**Keywords:** incidence, miR-146a, polymorphisms, prognosis, stroke

## Abstract

We conducted a case-control study investigating the association between the single-nucleotide polymorphism rs2910164 in microRNA (miR)-146a and the risk and prognosis of stroke. We recruited a total of 1139 ischemic stroke patients and 1585 sex- and age-matched control subjects. After a median follow-up period of 4.5 years, 1071 of these ischemic stroke patients were then recruited for a prospective study. Our study revealed that rs2910164 was not associated with ischemic stroke incidence (odds ratio = 1.00; 95% confidence interval (CI) = 0.80–1.24; *p* = 0.985) by multivariate logistic regression. Meta-analysis of our case-control study and three others on Asian populations also suggested that there was no relationship between rs2910164 and ischemic stroke incidence. The significance of differences in long-term outcomes was examined by the log-rank test of the respective comparison groups. The prospective study showed that rs2910164 led to a 1.56-fold increased risk of stroke recurrence (hazard ratio (HR) = 1.56; 95% CI = 1.10–2.20; *p* = 0.013) and a 2.13-fold increased risk of death caused by cardiovascular disease or stroke (Csdeath) (HR = 2.13; 95% CI = 1.31–3.46; *p* = 0.002). The independent association of rs2910164 with stroke prognosis was evaluated using Cox regression models. Therefore, rs2910164 appears to be a strong predictor of stroke prognosis but not of stroke incidence in Asian populations.

## 1. Introduction

Stroke has a limited therapeutic time window and a very high rate of recurrence, and thus is a leading cause of death and constitutes a heavy economic burden in many countries, including China [1,2,3,4]. It is a multifactorial disease affected by environmental and genetic risk factors including hypertension, diabetes mellitus, smoking, hyperlipidemia, and hyperhomocysteinemia [5,6]. Multiple susceptibility genes have been demonstrated to have a relationship with an enhanced risk of stroke or worse stroke prognosis, including *F5*, angiotensin-converting enzyme (*ACE*), methylenetetrahydrofolate reductase (*MTHFR*), serpin peptidase inhibitor 1 (*SERPINE1*), apolipoprotein E (*APOE*) [7], cytochrome P450 2C19 (*CYP2C19*) [8], and platelet-derived growth factor D (*PDGF-D*) [9], as well as chromosome 12p13 variants [10]. However, the genetic factors identified cannot fully explain the observed inherited risk of stroke.

MicroRNAs (miRNAs) are a class of endogenous, small, ~22-nucleotide non-coding RNAs attached to the 3′-untranslated regions of mRNAs through highly conserved seed sequences, and are known to negatively regulate mRNA expression. Genetic alterations in miR sequences impact on precursor processivity, maturation, expression, and ultimately influence the expression of target mRNAs [11,12,13]. miRNAs are important in biological processes including cell differentiation, proliferation, growth, stress resistance, and metabolism, as well as the pathophysiology of neurodegenerative disease, cancer, and cardiovascular disease [14,15,16]. Emerging evidence also indicates that circulating miRNAs may be novel biomarkers for the diagnosis and prognosis of stroke [17,18]. This reflects their role in modulating transcriptional programs that affect the processes of atherosclerosis, including endothelial integrity, inflammation, and extracellular matrix remodeling [19,20].

An important and universal type of genetic variation are single-nucleotide polymorphisms (SNPs) [21], which may affect miRNA function by modulating biogenesis or target selection [22]. Recently, a well-documented common polymorphism in a pre-miRNA sequence (miR-146a C > G (rs2910164; chromosome 5, 160485411)) was found to be involved in a variety of diseases [23,24,25,26]. This variant altered the specific base pairing of the stem region, which influenced the expression of mature miR-146a [27]. This binds to target mRNAs including tumor necrosis factor-α (TNF-α) [28], C-reactive protein and interleukin-1 receptor-associated kinase-1 (IRAK1) [29], which affect vascular damage responses and inflammation-related atherosclerosis in the development of stroke. However, studies into the relationship between the miR-146a rs2910164 SNP and ischemic stroke in different ethnic populations have provided conflicting results.

To investigate this in greater detail, we designed a case-control study of ischemic stroke patients in a Chinese population and a meta-analysis of four case-control studies into the role of rs2910164 in Asian ischemic stroke patients. A prospective study was also conducted to evaluate the effects of rs2910164 on stroke prognosis.

## 2. Results

### 2.1. Baseline Characteristics

The baseline characteristics of the ischemic stroke patients and control groups are listed in Table 1. After a follow-up (median time, 4.5 years) of 1071 ischemic stroke patients, a total of 196 recurrent strokes were recorded. The CC, GC, and GG rs2910164 genotype frequencies were 31.2%, 54.3%, and 14.5% for patients, and 30.5%, 54.8%, and 14.7% for controls, respectively (Table 2). SNP genotype distributions in both groups followed the Hardy–Weinberg equilibrium (HWE).

### 2.2. rs2910164 Is Not Associated with Ischemic Stroke Incidence

Under the dominant model, the GG + CG genotype of rs2910164 was not associated with ischemic stroke incidence compared with the CC genotype (OR = 1.00, 95% CI = 0.78–1.27, *p* = 0.961) after adjustment for Model 3. For the cerebral thrombosis subgroup, no association between rs2910164 and ischemic stroke incidence (OR = 1.02, 95% CI = 0.84–1.24, *p* = 0.834) after adjustment for Model 3, as was the case for the lacunar infarct subgroup (OR = 0.96, 95% CI = 0.76–1.22, *p* = 0.745) (Table 3). The rs2910164 genotype was not connected with the ischemic stroke incidence according to sex after adjustment for Model 2 (Table 4).

Under the recessive model, the rs2910164 GG genotype was not associated with ischemic stroke incidence compared with the GC + CC genotype (OR = 1.00, 95% CI = 0.80–1.24, *p* = 0.985) after adjustment for Model 3. For the cerebral thrombosis subgroup, no association between rs2910164 and ischemic stroke incidence (OR = 0.99, 95% CI = 0.77–1.28, *p* = 0.950) after adjustment for Model 3, as was the case for the lacunar infarct subgroup (OR = 1.02, 95% CI = 0.75–1.38, *p* = 0.922) (Table 3). The rs2910164 genotype was not associated with the ischemic stroke incidence according to sex after adjustment for Model 2 (Table 4).

### 2.3. Meta-Analysis of the Relationship between rs2910164 and Ischemic Stroke Incidence

**Figure 1:**
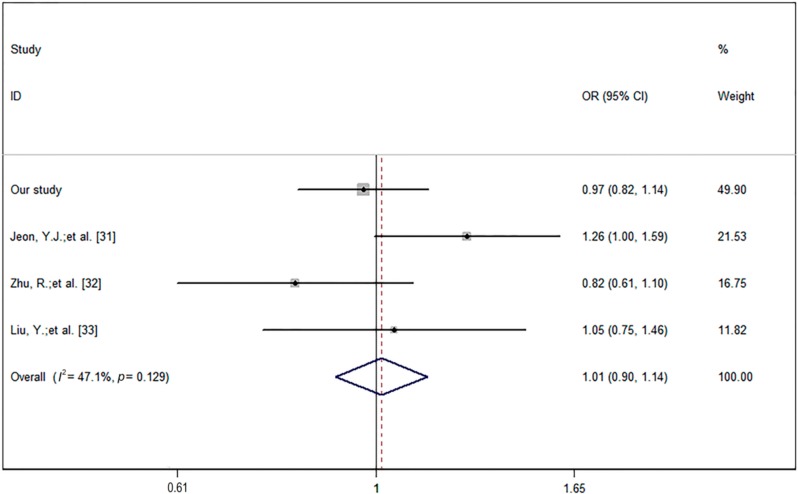
Forest plot showed the relationship between rs2910164 and ischemic stroke in Asian populations (four studies) under the dominant model (genotype (GG + CG) *vs.* CC). Heterogeneity: χ^2^ = 5.67, *p* = 0.129, *I*^2^ = 47.1%.

A forest plot was constructed from the findings of four studies showing the relationship between rs2910164 and ischemic stroke in Asian populations under the dominant model (genotype (GG + CG) *vs.* CC) (Figure 1). This suggested that rs2910164 does not affect the ischemic stroke incidence (OR = 1.01, 95% CI = 0.90–1.14).

### 2.4. rs2910164 Is a Strong Predictor of Stroke Prognosis

**Figure 2:**
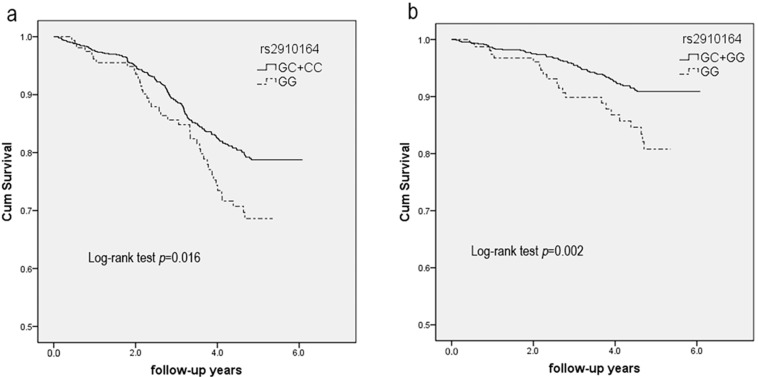
Association between rs2910164 polymorphism and stroke recurrence and Csdeath under the recessive model of inheritance. (**a**) Effect of the variant rs2910164 on stroke recurrence in patients with ischemic stroke. Kaplan–Meier estimates of the cumulative recurrence–free probability in ischemic stroke patients based on the C allele of rs2910164 polymorphism (log-rank statistic χ^2^ = 5.796, *p* = 0.016); (**b**) Effect of the variant rs2910164 on Csdeath in ischemic stroke patients. Kaplan–Meier estimates of the cumulative event–free survival probability in ischemic stroke patients based on the C allele of rs2910164 polymorphism (log–rank statistic χ^2^ = 9.155, *p* = 0.002).

This prospective study showed that the rs2910164 GG genotype notably increased the risk of stroke recurrence (Figure 2a, *p* = 0.016). Kaplan–Meier estimates of the cumulative recurrence-free probability in ischemic stroke patients based on the C allele of rs2910164 polymorphism were: log–rank statistic χ^2^ = 5.796; *p* = 0.016. Cox proportional hazards analysis indicated that the GG genotype had a 1.56-fold increased risk for recurrence (Table 5, HR = 1.56, 95% CI = 1.10–2.20, *p =* 0.013) after adjusting for Model 3. Additionally, the GG genotype significantly increased the risk of death caused by cardiovascular disease or stroke (Csdeath) (Figure 2b, *p* = 0.002). Kaplan–Meier estimates of the cumulative event-free survival probability in ischemic stroke patients based on the C allele of rs2910164 polymorphism were: log-rank statistic χ^2^ = 9.155, *p* = 0.002. The analysis of Cox proportional hazards showed that the GG genotype had a 2.13-fold increased risk for Csdeath (Table 5, HR = 2.13, 95% CI = 1.31–3.46, *p* = 0.002) after adjusting for Model 3.

## 3. Discussion

In this study, we investigated the association of the miR-146a rs2910164 SNP with stroke incidence and prognosis. Our large-scale prospective investigation showed that rs2910164 was associated with a 1.56-fold increased risk of stroke recurrence and a 2.13-fold increased risk of Csdeath in a Chinese population. However, we observed no association with ischemic stroke incidence. To the best of our knowledge, it is the first time we found the association between rs2910164 with ischemic stroke prognosis.

There are a number of possible mechanisms to explain why rs2910164 is a significant predictor of stroke prognosis. rs2910164 involves a C-to-G nucleotide substitution, which can cause a C:U pair change to a G:U mismatch in the stem structure of the miR-146a precursor, and therefore decreases the expression of mature miR-146a. miR-146a regulates a pathway and it can accelerate the binding of the transcriptional repressor RelB to the *TNF-α* promoter [28]. miR-146a primarily targets *IRAK1* and *TRAF6*, resulting in the inhibition of nuclear factor (NF)-κB via the Toll-like receptor pathway [29]. Therefore, the down-regulation of miR-146a may increase inflammation-related atherosclerosis and affect vascular damage response by increasing the levels of TNF-α, TRAF6, and IRAK1. Overexpression of miR-146a in peripheral blood mononuclear cells activates Th1 cells and induces the expression of TNF-α, monocyte chemotactic protein 1, NF-κB, and p65 through post-transcriptional enhancement of the T-bet pathway [30].

Previously, rs2910164 was significantly associated with ischemic stroke prevalence and increased stroke risk in female, normotensive, and nondiabetic groups in a South Korean population [31]. However, we found no association between rs2910164 and ischemic stroke incidence. A previous investigation by Zhu *et al.* observed that rs2910164 had a protective role against the incidence of large-artery atherosclerotic stroke in the northern Chinese Han population [32], although Liu *et al.* failed to find any relationship between rs2910164 and ischemic stroke [33]. These different findings could reflect ethnic variations and limitations of sample sizes, but most important, the sex, subtype of stroke may contribute a lot to this. For this reason, we have analyzed the association between rs2910164 and ischemic stroke incidence based on stroke subtype and sex, and we conducted a meta-analysis of the four above mentioned studies, but the result showed no relationship between rs2910164 and ischemic stroke incidence.

The current study has a number of limitations. Although the sample size was relatively large, a larger patient population involving other ethnicities should be included. Additionally, in our meta-analysis, the absence of original data restricted us to further evaluate gene–gene and gene-environment interactions. Independent genetic studies should also be performed to detect levels of mature miR-146a and to elucidate novel target genes and regulatory molecules. In addition, we performed analyses for several genetic models, namely, dominant, recessive, codominant, and additive ones; the results showed that the dominant model and recessive model are the most appropriate.

## 4. Materials and Methods

### 4.1. Study Population

Patients in the case-control study were recruited from among participants of the previously described Multicenter Chinese Stroke Study [34]. Between November 2000 and November 2001, we consecutively recruited 1139 ischemic stroke patients from seven clinical centers (Beijing, Tianjin Yanzhou, Xi’an, Wuhan Xiehe, Wuhan Tongji, and Chongqing, China) together with 1585 age- and sex-matched control subjects. To minimize phenotypic heterogeneity, we recruited patients with only one of the two subtypes of ischemic stroke (cerebral thrombosis and lacunar infarct).

We used neurological examination, magnetic resonance imaging (MRI), or computed tomography (CT) to confirm stroke, in strict accordance with the criteria of the International Classification of Diseases (9th revision). It was defined as a sudden onset of nonconvulsive and focal neurological deficits persisting for >24 h. We excluded patients who had other kinds of stroke: subarachnoid hemorrhage, cerebrovascular malformation, embolic brain infarction, brain tumors, and transient ischemic attack, and other comorbidity diseases such as: inflammation, collagenosis, liver, metabolic disease, tumorous, or renal disease.

Our investigations were conducted consistent with the rules of the Declaration of Helsinki, and identified by the ethics committee and institutional review board of Fuwai Hospital. All participants were given written informed consent.

### 4.2. Follow-up and Outcome Assessment in a Prospective Study

Of the 1139 ischemic stroke patients, 1071 were recruited after an average 4.5 years of follow-up until 2006 by physicians through the administration of a standard questionnaire and telephone contact. Endpoints included stroke recurrence and Csdeath. Recurrent stroke was defined as a new-onset acute new focal neurological deficit without obvious cause other than vascular disease origin after stroke or an acute aggravation of the existing focal neurological deficit without obvious cause other than vascular disease which was occurring at least 21 days after the first stroke [35]. Deaths were reported by family members. No significant differences in the frequency of genotypes and clinical parameters of patients were found between those who were followed up and those who were lost to follow-up. The event-free group comprised those patients who had no events and were unable to be followed up completely.

### 4.3. Measurements of Biochemical Parameters and Collection of Clinical Data

Routine clinical interviews were conducted to ascertain each patient’s history of hypertension, diabetes mellitus, cigarette smoking, and alcohol intake. The 12-h overnight fasting blood of these patients were collected. In patients with acute medical events, we delayed the collection of blood samples by six weeks. Plasma was separated by centrifugation and the white blood cell buffy coat was stored at −80 °C. Biochemical variables, including total plasma cholesterol, triglyceride, high-density-lipoprotein cholesterol levels, and blood glucose, were determined by an automatic Hitachi 7060 chemistry analyzer (Hitachi, Tokyo, Japan).

### 4.4. Genotyping Variants of rs2910164

Ligase detection reaction (LDR) was used to genotype rs2910164 variants (Shanghai Biowing Applied Biotechnology Co., Ltd: Shanghai, China). Primer or probe sequences and PCR or LDR product lengths of the variants are listed in Table 6. Fragment was amplified in 20-µL multiplex PCR reaction mixtures including 50 ng (1 µL) of genomic DNA, 2 µL of dNTPs,2 µL of 1× buffer, 0.6 µL of Mg^2+^, 0.2 µL of Taq polymerase, 4 µL of 1× Q-solution, 0.4 µL of primer mix, and 9.8 µL of ddH_2_O, using the thermal cycler Gene Amp PCR system 9600 (Perkin Elmer, Waltham, MA, USA). Further amplification was conducted in 10-μL multiplex LDR reaction mixtures containing 100 ng of original PCR product (1 µL), 0.05 μL of NEB Taq DNA ligase ,1 μL of probe mix, and 6.95 μL of ddH_2_O, using the ABI PRISM 377 DNA Sequencer detected and GeneMapper software (ABI) analyzed.

### 4.5. Statistical Analysis

The χ^2^ test was performed to test genotype and allele frequencies, qualitative variables, and HWE. Multivariable logistic regression model was conducted to evaluate associations between rs2910164 and stroke incidence. In the prospective study, Kaplan–Meier survival analysis and Cox proportional hazards models were used to describe the association between rs2910164 and stroke recurrence and prognosis. The unadjusted hazard ration was shown in Model 1. In Model 2, age and sex were adjusted, and age, sex, hypertension, diabetes mellitus, smoking status, and alcohol intake were adjusted in Model 3. Two-tailed *p* < 0.05 was considered significant. Statistical analyses were carried out with SPSS software, version 13.0 (SPSS Inc., Chicago, IL, USA).

### 4.6. Meta-Analysis

A meta-analysis of four studies on the role of rs2910164 in ischemic stroke, including our case-control study, two other studies in Chinese populations, and one in a South Korean population, was performed (Table 7). A total of 2481 ischemic stroke patients and 2910 controls were analyzed. Literature was searched in PubMed and EMBASE databases, using the following retrieval strategy: miR-146a AND polymorphisms AND stroke incidence. The inclusion criteria are as followed: (1) the association between the miR146a rs2910164 polymorphism and ischemic stroke are case-control studies; (2) containing original data; and (3) following HWE. Exclusion criteria were: (1) study design other than case-control; (2) not reporting genotypic and allelic frequencies; and (3) family members studied based on linkage considerations. Two investigators discussed to resolve the disagreements between them. We calculated odds ratio (OR), 95% confidence interval (95% CI) through the fixed effects model or DerSimonian–Laird and random effects model to describe the relationship between rs2910164 and stroke incidence. Statistical analysis was carried out using Stata (9th edition; Stata Corporation, College Station, TX, USA) and RevMan (5th edition) software.

## 5. Conclusions

We found that rs2910164 increased the risk of stroke recurrence and Csdeath in a Chinese population but did not predict stroke incidence in Asian populations, suggesting that it has the potential to be a target for therapeutic interventions that aim to reduce inflammation and improve stroke outcome.

## Figures and Tables

**Table 1:** Baseline characteristics of the study population.

Characteristics	Control (*n* = 1585)	Ischemic Stroke (*n* = 1139)	*p*-Value
Age (years)	59.50 ± 8.50	61.30 ± 9.40	<0.001
Male (%)	903 (57)	718 (63)	0.004
SBP, mmHg	128.86 ± 17.68	145.43 ± 22.26	<0.001
DBP, mmHg	79.55 ± 9.79	86.24 ± 12.36	<0.001
TC, mmol/L	5.17 ± 1.29	5.07 ± 1.20	0.620
TG, mmol/L	1.56 ± 1.15	1.90 ± 1.56	<0.001
HDL-C, mmol/L	1.44 ± 0.42	1.23 ± 0.44	<0.001
Glucose, mmol/L	5.69 ± 1.83	6.28 ± 2.96	<0.001
Cigarette smoking (%)			
Never	992 (62.60)	584 (51.30)	<0.001
Former	201 (12.70)	251 (22.00)	<0.001
Current	392 (24.70)	304 (26.70)	<0.001
Alcohol intake (%)			
Nondrinker	1453 (91.7)	936 (82.2)	<0.001
Drinker	132 (8.3)	203 (17.8)	<0.001
Hypertension history (%)	412 (26.00)	714 (62.70)	<0.001
DM history (%)	86 (5.40)	177 (15.50)	<0.001

SBP: Systolic blood pressure; DBP: Diastolic blood pressure; TC: Total plasma cholesterol; TG: Triglyceride; HDL-C: High-density-lipoprotein cholesterol; DM: Diabetes mellitus.

**Table 2:** Genotype and allele frequencies of rs2910164.

Genotype	No. of Controls (%)	No. of Strokes (%)	*p-*Value
C/C	483 (30.5)	355 (31.2)	
G/C	869 (54.8)	618 (54.3)	
G/G	233 (14.7)	166 (14.5)	
Total	1585 (100)	1139 (100)	0.928

**Table 3:** rs2910164 genotype was not associated with ischemic stroke incidence.

Variant Group (Number)	Genotype Number (%)		Model 1		Model 2		Model 3	
			OR (95% CI)	*p*-Values	OR (95% CI)	*p*-Values	OR (95% CI)	*p*-Values
**Dominant model**	**CC**	**GG + CG**						
Control (1585)	483 (30.5)	1102 (69.5)	1.00		1.00		1.00	
Stroke (1139)	355 (31.2)	784 (68.8)	1.03 (0.81–1.31)	0.801	0.98 (0.77–1.25)	0.870	1.00 (0.78–1.27)	0.961
Cerebral thrombosis (718)	223 (31.1)	495 (68.9)	0.97 (0.80–1.18)	0.778	1.03 (0.84–1.25)	0.792	1.02 (0.84–1.24)	0.834
Lacunar infarct (421)	132 (31.4)	289 (68.6)	0.96 (0.76–1.21)	0.728	0.96 (0.76–1.21)	0.707	0.96 (0.76–1.22)	0.745
**Recessive model**	**GC + CC**	**GG**						
Control (1585)	1352 (85.3)	233 (14.7)	1.00		1.00		1.00	
Stroke (1139)	973 (85.4)	166 (14.6)	0.99 (0.80–1.23)	0.927	1.02 (0.82–1.27)	0.868	1.00 (0.80–1.24)	0.985
Cerebral thrombosis (718)	613 (85.4)	105 (14.6)	1.01 (0.78–1.29)	0.962	0.97 (0.75–1.25)	0.813	0.99 (0.77–1.28)	0.950
Lacunar infarct (421)	360 (85.5)	61 (14.5)	0.98 (0.73–1.33)	0.913	0.99 (0.73–1.35)	0.971	1.02 (0.75–1.38)	0.922

Abbreviations: OR: Odds ratio; CI: Confidence interval. Model 1: Unadjusted OR; Model 2: Adjusted for age and sex; Model 3: Adjusted for age, sex, hypertension, diabetes mellitus, smoking status, and alcohol intake (adjusted ORs (95% CI) and adjusted *p*-values were computed using multivariate logistic regression analyses).

**Table 4:** rs2910164 genotype was not associated with ischemic stroke incidence according to sex.

Variant Group	Model 1		Model 2	
	OR (95% CI)	*p*–Value	OR (95% CI)	*p*–Value
**Dominant model**				
Sex				
Male	0.81 (0.65–1.00)	0.049	0.84 (0.68–1.04)	0.109
Female	1.25 (0.96–1.63)	0.097	1.31 (1.00–1.73)	0.052
**Recessive model**				
Sex				
Male	0.97 (0.74–1.28)	0.840	0.97 (0.73–1.28)	0.821
Female	1.07 (0.76–1.50)	0.709	1.05 (0.74–1.50)	0.771

Abbreviations: OR: Odds ratio; CI: Confidence interval. Model 1: Unadjusted OR; Model 2: Adjusted for age, hypertension, diabetes mellitus, smoking status, and alcohol intake (adjusted ORs (95% CI) and adjusted *p* values were computed using multivariate logistic regression analyses).

**Table 5:** rs2910164 GG genotype increased risk of stroke recurrence and Csdeath.

Genotype	No. of Patients	No. of Events	Model 1		Model 2		Model 3	
			HR (95% CI)	*p*–Value	HR (95% CI)	*p*–Value	HR (95% CI)	*p*–Value
**Recurrence**								
CC + GC	912	156	1.00	0.017	1.00	0.014	1.00	0.013
GG	159	40	1.53 (1.08–2.16)		1.54 (1.09–2.18)		1.56 (1.10–2.20)	
**Csdeath**								
CC + GC	912	63	1.00	0.003	1.00	0.003	1.00	0.002
GG	159	22	2.08 (1.28–3.38)		2.11 (1.30–3.42)		2.13 (1.31–3.46)	

Abbreviations: HR, hazard ratio; CI, confidence interval. Model 1: unadjusted HR; Model 2: adjusted for age and sex; Model 3: adjusted for age, sex, hypertension, diabetes mellitus, smoking status, and alcohol intake (adjusted HRs (95% CI) and adjusted *p*-values were computed using Cox regression analyses).

**Table 6:** Primer or probe sequences and PCR or LDR product length of rs2910164.

Variants	Primer or Probe	Sequence (5′–3′)	PCR or LDR
rs2910164G/C	rs2910164–up	CTGGACTGCAAGGAGGGGTCTT	151
	rs2910164–low	GTCCTCAAGCCCACGATGACAG	
	rs2910164_modify	P–TGAAATTCAGTTCTTCAGCTGGGATTT–FAM	
	rs2910164_G	TTCCGCGTTCGGACTGATATCATGGGTTGTGTCAGTGTCAGACATG	94
	rs2910164_C	TACGGTTATTCGGGCTCCTGTCATGGGTTGTGTCAGTGTCAGACATC	95

Abbreviations: P, phosphorylated; FAM, Carboxyfluorescein.

**Table 7:** Association between rs2910164 polymorphism and ischemic stroke incidence in Asian populations (four studies).

Study	Case	Control	OR, 95%CI
Our study	1139	1585	0.97 (0.82–1.14)
Jeon, Y.J.; *et al.* [31]	678	553	1.26 (1.00–1.59)
Zhu, R.; *et al.* [32]	368	381	0.82 (0.61–1.10)
Liu, Y.; *et al.* [33]	296	391	1.05 (0.75–1.46)
Total	2481	2910	1.01 (0.90–1.14)

Heterogeneity: χ^2^ = 5.67, *p* = 0.129, *I*^2^ = 47.1%.
